# The role of media in shaping pro-environmental behaviors: integrating media system dependency theory and norm activation theory

**DOI:** 10.3389/fpsyg.2025.1520537

**Published:** 2025-07-02

**Authors:** Chi-Horng Liao

**Affiliations:** Bachelor Program in Digital Media and Technology, Tzu Chi University, Hualien City, Taiwan

**Keywords:** pro-environmental behavior (PEB), media system dependency theory (MSDT), norm activation theory (NAT), fuzzy decision-making trial and evaluation laboratory (F-DEMATEL), structural equation modelling (SEM), Self-efficacy, Personal Norm

## Abstract

This study examines how media factors influence pro-environmental behavior (PEB) through an integration of Media System Dependency Theory (MSDT) and Norm Activation Theory (NAT). Using both Fuzzy Decision-Making Trial and Evaluation Laboratory (F-DEMATEL) and Structural Equation Modeling (SEM), it evaluates the effects of media factors on NAT components—awareness of consequences and ascription of responsibility—and their role in activating personal norms that drive PEB. The study also considers the moderating impact of self-efficacy on the link between personal norms and PEB. Findings from F-DEMATEL, based on input from 20 experts, indicate a causal connection between media factors (exposure, attention, dependence, credibility) and NAT variables. SEM analysis from a large sample of 1,594 participants in Taiwan reveals that media factors influence NAT components, with awareness of consequences and ascription of responsibility indirectly affecting PEB via personal norms. However, the direct effects of the ascription of responsibility and personal norms and the awareness of consequences and PEB were not significant, differing from prior research. Additionally, self-efficacy was found to moderate the positive influence of personal norms on PEB. This study contributes theoretical and practical insights, discusses limitations, and offers directions for future research. This research contributes theoretically and practically, highlights limitations, and suggests avenues for future investigation.

## 1 Introduction

Despite the widespread global aspiration for a clean and thriving ecosystem, the world faces escalating environmental challenges, including climate change, rising temperatures, and the detrimental impacts of air and water pollution (Onwezen et al., 2013). Researchers attribute most of these issues to human activities (Grilli and Curtis, 2021). Despite efforts to address environmental problems in Taiwan, unresolved issues persist alongside some successes. Global challenges such as acid rain, escalating greenhouse gas emissions, and contaminated water systems coexist with local concerns influenced by geographical and natural factors (Grano, 2015). The accelerated economic growth and urbanization in Taiwan have led to increased waste production, polluting soil, water, and air with varying threats to human health (Grano, 2015). Rapid population growth has significantly reduced Taiwan’s natural forests, comprising over half of the island’s total land area. Additionally, constructing roads and railways has exacerbated accessibility issues in remote mountain regions. To mitigate these environmental challenges, human intervention and behavior change are imperative. People must assume responsibility for addressing climate change, engaging in various environmental actions such as modifying consumption behavior, and actively participating in environmental advocacy to contribute to environmental conservation and protection (Ho et al., 2015).

Numerous studies have proposed various channels to promote the adoption of environmentally responsible behaviors, focusing on how knowledge impacts individuals’ engagement in pro-environmental actions. Mass media has been identified as a powerful tool in increasing environmental awareness and encouraging participation in behaviors supporting the environment (Arlt et al., 2011). Media coverage and campaigns have shifted people’s perception of nature, emphasizing their role as stewards rather than controllers, thereby influencing their attitudes and behaviors towards environmental responsibility (Wang, 2022). While existing research has explored the relationship between environmental awareness and pro-environmental actions, it has often overlooked the role of mass media in shaping individual behavior change by fostering awareness of consequences and ascription of responsibility. Additionally, there needs to be more understanding of the acquisition and linkage of personal norms that can significantly impact pro-environmental behaviors.

Individuals’ belief in the morality or correctness of pro-environmental actions influences their perception, especially considering the additional resources and efforts required for such behaviors (Truelove and Gillis, 2018). According to Confente and Scarpi (2021), the Norm-Activation Theory (NAT) has been widely employed in various studies to understand pro-environmental actions, intentions, and behaviors, showing efficacy in contexts like car usage and overall pro-environmental behaviors (Eriksson and Forward, 2011; Nordlund and Garvill, 2003). However, specific gaps persist in applying NAT, notably the need for more consideration for self-efficacy and ascription of responsibility in earlier research (Onwezen et al., 2013). While the emphasis has been on awareness of consequences (Steg and Nordlund, 2018), this study addresses the initial NAT elements, awareness of consequences and ascription of responsibility, as stimulators of personal norms. Additionally, self-efficacy is examined as a moderator in the relationship between personal norms and pro-environmental behaviors, acknowledging that personal norms are more influential when individuals believe in their ability to contribute to environmental problem-solving. Thus, the study utilizes NAT to elucidate personal norms and pro-environmental behaviors, aiming to fill gaps in the theory regarding variables like self-efficacy and ascription of responsibility.

The study incorporates the Media System Dependency Theory (MSDT) to investigate the influence of media factors (media exposure, media attention, media dependency, and media credibility) on personal norms and pro-environmental behaviors (PEB). MSDT, which explores the impact of dependency on cognitive, affective, and behavioral changes, is employed to understand how individuals’ reliance on mass media for environmental information affects their norms and behaviors, especially during uncertain times and societal upheavals. The study integrates MSDT and the Norm-Activation Theory (NAT) to predict PEB in Taiwan, addressing gaps in previous research. The fuzzy decision-making trial and evaluation laboratory (F-DEMATEL) method and structural equation modeling (SEM) examine how media elements influence awareness of consequences and ascription of responsibility, triggering personal norms and affecting individuals’ environmentally conscious actions. F-DEMATEL is chosen for its two-sided approach, benefiting the analysis of complex systems, while SEM allows for hypothesis testing and simultaneous estimation of variables. The study employs a questionnaire survey and nonprobability sampling to collect data from adults in Taiwan. Additionally, it explores the indirect effects of media factors on PEB through NAT personality trait elements. It examines the moderating role of self-efficacy in the relationship between personal norms and PEB, a novel aspect of the research.

The present study offers significant contributions to both the academic literature and practical applications. First, by integrating Media System Dependency Theory (Ball-Rokeach and DeFleur, 1976) and Norm Activation Theory (Schwartz, 1977), it bridges communication and psychological frameworks, thereby advancing theoretical understanding of the mechanisms through which media influence environmental behavior. This integration moves beyond the traditional view of media as mere channels of information transmission, highlighting their role in activating personal norms. Whereas prior research has often treated these theories in isolation, this study synthesizes them to uncover the complex cognitive and emotional pathways that drive pro-environmental action. Second, it extends NAT by demonstrating that media particularly digital and social media can function as contextual cues for norm activation, an area that has received limited attention in previous scholarship (Han, 2015b). Third, the study contributes to the fields of environmental psychology and communication studies by conceptualizing media as a structural force that shapes ecological awareness, moral responsibility, and behavioral intentions (De Groot and Steg, 2009). Finally, it proposes a framework for designing environmental campaigns that strategically utilize media platforms to activate personal norms through emotional appeals, heightened moral responsibility, and increased problem awareness.

The objectives of the study include (1) examining the relationships between media factors and NAT personal trait elements, as well as how they influence personal norms, (2) exploring how media factors indirectly impact PEB, (3) investigating the role of personal norms in mediating the relationship between NAT elements (awareness of consequences and ascription of responsibility) and PEB, and (4) testing the influence of self-efficacy in moderating the relationship between personal norms and PEB.

## 2 Literature review and hypotheses development

### 2.1 Theoretical background

#### 2.1.1 The media system dependency theory (MSDT)

In 1974, Ball-Rokeach introduced the foundational concepts of the Media System Dependency Theory (MSDT), a social theory focused on mass media (Kim, 2020). MSDT posits that individuals rely more on mass media for information, especially when alternative sources are available (Loges, 1994). This dependency on media is linked to changes in attitudes and behavior, emphasizing the media’s role in controlling essential resources for individuals to achieve their goals (Zhang and Zhong, 2020). The theory asserts that media impact is heightened during periods of instability, uncertainty, or ambiguity, as well as in advanced media systems providing abundant information (Groshek, 2011; Loveless, 2008; Matei and Ball-Rokeach, 2003). Media dependency increases attention and the likelihood of message effects, intentional or unintentional, under these circumstances (Zhang and Zhong, 2020).

MSDT has been applied to study democratization processes, social movements, and pro-environmental behavioral intentions (Groshek, 2011; Ho et al., 2015). The theory encompasses two main components: media use and media credibility. Media credibility is closely tied to the broader social system, particularly political institutions, and the public’s evaluation of media credibility is intertwined with social trust (Li et al., 2007). The media significantly influences individuals’ actions regarding the environment, as people rely on it to receive messages about environmental issues, ultimately impacting their environmental behavior. However, further exploration is needed to comprehend the specific effects of media usage on individuals’ environmental behavior.

#### 2.1.2 Norm activation theory (NAT)

The NAT was initially developed by Schwartz (1977) in the context of altruistic behavior. Personal norms form the core of this model. Schwartz (1977) states that these norms are actively experienced as feelings of moral obligation, not just intentions. NAT holds that personal norms have a guiding effect on behavior; if an individual believes he or she has moral obligations to protect the environment, they will adopt corresponding behaviors (Klöckner, 2013). This theory particularly emphasizes the activation conditions of personal norms, namely awareness of consequences and ascription of responsibility (Harland et al., 2007), because inactive norms are challenging to influence individual behavior. Many researchers have applied NAT to explain environmentally significant behavior with promising results, showing that NAT variables influence PEB as it focuses on the moral drivers of PEB (Klöckner, 2013).

#### 2.1.3 Pro-environmental behavior (PEB)

According to Huang (2016), the definition of PEB can be determined by its impact and purpose. When we consider the impact of environmental behavior, it entails actions that have the potential to create positive changes in the availability of resources, energy, or ecological interactions. On the contrary, if we focus on intent, PEB denotes actions carried out to alter the environment from the perspective of the individual or group involved (Stern, 2000). Environmental research primarily focuses on the impact of environmental behavior on individuals, such as recycling, conserving resources, and adopting environmentally friendly habits. Stern (2000) categorized PEB into public-sphere and private-sphere environmentalism, with public-sphere environmentalism involving active engagement in environmental groups and demonstrations and private-sphere environmentalism involving non-active actions like buying items and managing waste disposal (Huang, 2016). This research examines private-sphere environmentalism, which falls within the impact-oriented definition of PEB.

### 2.2 Hypotheses development

#### 2.2.1 The impact of media exposure on awareness of consequences and ascription of responsibility

Media exposure is a crucial factor in evaluating the effectiveness of media campaigns. It refers to the media content people consume on specific topics (Slater et al., 2009; Wang et al., 2024), such as environmental preservation, healthcare, and politics (Camaj and Weaver, 2013; Li et al., 2017). Awareness of consequences is an individual’s ability to recognize and react to environmental cues indicating the need for action (Liu et al., 2017). When individuals become aware of the consequences of their actions, they reflect on their responsibility for causing problems and consider their ability to contribute to finding solutions. The first step towards taking responsible actions is becoming conscious of their challenges, which influences their personal norms and motivation to act (Davis et al., 2015; Jakovcevic and Steg, 2013). In this study, awareness of consequences refers to the tendency to become aware of the negative consequences of environmental destruction.

Mass media exposure can enhance individuals’ understanding of societal issues, increasing awareness and motivation to engage in actions related to these concerns (Lee, 2015; Li et al., 2017; Manickam, 2014). For instance, exposure to environmental information can lead to a deeper understanding of environmental values and knowledge. Previous studies, for example (Ando et al., 2021; Trivedi et al., 2018; Wang et al., 2022), have shown that mass media exposure or campaigns can effectively affect awareness of consequences, increasing concern and knowledge about important environmental issues. Moreover, Rahman et al. (2024) demonstrated that social media campaigns highlighting the dangers of misinformation effectively enhanced users’ awareness of its broader societal implications, including threats to public health and the integrity of democratic processes. In a similar vein, Kim et al. (2023) reported that sustained exposure to climate-related news content on digital platforms significantly heightened young adults’ understanding of the environmental consequences associated with consumer behavior. Therefore, based on the above evidence, we proposed the following hypothesis:

Hypothesis 1a: Media exposure to environmental information by the public positively affects awareness of consequences.

Regarding the ascription of responsibility, NAT refers to the level of responsibility a person feels for the environmental consequences resulting from human actions (Harland et al., 2007; Liu et al., 2017). In the present study, the ascription of responsibility refers to an individual’s natural inclination to view themselves as accountable for the potential adverse outcomes related to environmental harm. The use of mass media campaigns is a standard method to address environmental concerns by bringing attention to the public and instilling a sense of responsibility towards these issues (Wang et al., 2022). These campaigns aim to increase awareness of environmental problems and promote a collective commitment towards their resolution (Sampei and Aoyagi-Usui, 2009). If someone is aware that their actions negatively impact others and the environment, they may feel accountable for these effects. Thus, they might believe that their eco-friendly behavior can help alleviate environmental problems and motivate them to follow their own set of personal norms (Steg and De Groot, 2010). In this regard, the study proposed the following hypothesis.

Hypothesis 1b: Media exposure to environmental information by the public positively affects the ascription of responsibility.

#### 2.2.2 The impact of media attention on awareness of consequences and ascription of responsibility

Media Attention, characterized by its inherent focus on communication, plays a crucial role, particularly in news consumption, where people naturally prioritize topics like environment, politics, sports, celebrities, crime, and accidents (Slater et al., 2009). Gong et al. (2022) defines media attention as the visibility or utilization of specific channels such as TV, print media, the web, or social platforms. Individuals instinctively concentrate their mental efforts on particular types of media communications (Slater et al., 2009). The significance of media coverage in shaping messages has increased, with research demonstrating a clear link between the quantity of media attention and behavioral modification (Karimi et al., 2021). In today’s world, the media is a critical source of substantial environmental information, expected to be the primary channel for those seeking information on issues like the greenhouse effect, climate change, ozone layer depletion, water and air pollution, and other hazards (Rosenthal, 2022).

Previous studies highlight the influential role of environmental news in traditional media in significantly raising awareness and comprehension within diverse communities (Karimi et al., 2021). Lee (2011) contends that focusing on environmental concerns compels the public to value engagement in environmental activities, increasing their inclination to participate. Media messages supporting the environment involve reporting news and launching campaigns to engage the public to raise awareness and encourage the adoption of Pro-Environmental Behaviors (PEB). Individuals with a higher interest in environmental news engage in extensive processing, gaining a deeper understanding, and paying attention to campaign messages can enhance the likelihood of achieving persuasive impacts (Ho et al., 2015). Furthermore, Meng et al. (2023) investigated the influence of social media on climate change awareness in China and determined that increased media exposure substantially enhanced users’ comprehension of climate-related impacts. This, in turn, fostered more environmentally conscious attitudes and behaviors. Similarly, Mohammed et al. (2023) analyzed media coverage during the COVID-19 pandemic, concluding that consistent and targeted reporting significantly heightened public awareness of health risks and promoted the adoption of precautionary measures. The above evidence led to the following proposed hypotheses.

Hypothesis 2a: Media attention to environmental information by the public positively affects awareness of consequences.

Hypothesis 2b: Media attention to environmental information by the public positively affects the ascription of responsibility.

#### 2.2.3 The impact of media dependency on awareness of consequences and ascription of responsibility

Dependency is a relationship where one party relies on another for resources to fulfill their needs or achieve goals (Zhang and Zhong, 2020). MSDT suggests that people rely more on mass media for information when faced with uncertainty and societal disruptions, such as natural catastrophes (Gong et al., 2022; Kim, 2020; Ho et al., 2015). The more the media functions to satisfy specific goals, the more dependent individuals are on it (Hussain et al., 2019). The media’s coverage of contradicting perspectives and news frames has contributed to public uncertainty about the causes and effects of climate change (Schuldt et al., 2011). However, extensive media attention to environmental issues can create a perception of risk within society by promoting public awareness about the consequences of climate change and other environmental challenges (Hansen, 2011). Consequently, the following hypothesis is proposed:

Hypothesis 3a: Media dependency on environmental information by the public positively affects awareness of consequences.

Hypothesis 3b: Media dependency on environmental information by the public positively affects the ascription of responsibility.

#### 2.2.4 The impact of media credibility on awareness of consequences and ascription of responsibility

Media credibility, defined by Huh et al. (2004), pertains to an audience’s perceived trustworthiness of media as a communication channel, emphasizing media outlets delivering messages over communication sources and the messages themselves (Kiousis, 2001). This concept is crucial for understanding the impacts of mass communication platforms, such as news outlets and advertising. Various studies confirm diverse credibility levels across media types, where high credibility leads to positive audience reactions (Huh et al., 2004; Jo, 2005). Media credibility significantly shapes individuals’ opinions and responses to information, regardless of content similarity. Furthermore, studies have demonstrated that credibility can enhance comprehension, as demonstrated in research related to climate change and health communication, where credible sources typically result in improved public awareness and readiness (Kiousis, 2001). Trusted sources prompt individuals to take environmental consequences seriously, while distrust may lead to reduced concern. Credible media sources are considered reliable and precise, and when they cover environmental issues, the information is treated seriously, enhancing public awareness (Lee and Cho, 2020). The credibility of media sources plays a pivotal role in shaping public perspectives on environmental matters, influencing opinions based on information source reliability. Therefore, the perceived trustworthiness of media outlets contributes significantly to a more informed and enlightened public, fostering greater understanding and empathy for environmental concerns. In this context, the following hypothesis is proposed.

Hypothesis 4a: Media credibility concerning environmental issues positively affects awareness of consequences.

The credibility of the media in presenting environmental issues is crucial for the ascription of responsibility. Credible sources present environmental matters in a trustworthy and impartial way, allowing individuals to attribute responsibility to entities like corporations, governments, or individuals (Lee and Cho, 2020). They provide accurate and extensively investigated information about factors and individuals responsible for environmental issues. As MSDT suggests, media’s influence is heightened in times of uncertainty, shaping decision-making processes. Environmental deterioration can lead to unstable conditions, endangering people’s livelihoods and lives (Zhang and Zhong, 2020). Credible media can motivate people to take responsibility for environmental issues, especially in poorer conditions. Accurate information helps assign responsibility efficiently, identifying responsible entities or individuals (Rosenthal, 2022). Trustworthy reporting contributes to precisely understanding environmental problems, impacting responsibility ascription, driving beneficial transformations, and ensuring accountability. This can drive beneficial transformations and ensure accountability in the face of environmental challenges. Hence, the following hypothesis is proposed.

Hypothesis 4b: Media credibility concerning environmental issues positively affects the ascription of responsibility.

#### 2.2.5 Awareness of consequences, the ascription of responsibility, and personal norms

Personal norms are an individual’s internal perception of their moral obligation to perform a particular action, often demonstrating a selfless perspective on human behaviors (Wu et al., 2022; Schwartz, 1977). Pro-environmental actions are often seen as selfless, aiming to improve living beings’ well-being or protect nature. Individuals develop stronger personal norms when they become aware of environmental issues and feel accountable for them, preventing attributing problems solely to others, industry, or government (Steg and Nordlund, 2018). Personal norms are considered robust when individuals believe their actions can address environmental issues, making them a crucial factor in shaping pro-environmental actions (Stern, 2000).

According to He and Zhan (2018), NAT highlights the importance of awareness of consequences and responsibility in influencing personal norms. It suggests that individuals take responsibility for their actions and are aware of potential outcomes, such as pollution, poor air quality, climate change, and floods (Setiawan, 2021). When a person is aware of the consequences, they recognize the negative outcomes that others or valuable things may face due to their lack of altruistic behavior (De Groot and Steg, 2009). This awareness leads to individuals recognizing the potential negative consequences of their actions, which can exacerbate problems (Lauper et al., 2016). As a result, individuals feel morally obligated to adopt pro-environmental actions, thereby reducing the negative impacts of their actions. Therefore, the following hypothesis is proposed:

Hypothesis 5a: Awareness of consequences positively and significantly relates to an individual’s personal norms.

Apart from the awareness of consequences, personal norms can also be triggered by the ascription of responsibility. According to Schwartz (1977), personal norms are activated in the NAT when awareness of consequences and ascription of responsibility are combined. Current research defines responsibility as an individual’s sense of responsibility for the harmful effects of their choices to engage in actions that harm the environment. As a result, individuals who recognize that their actions can adversely affect the environment experience a feeling of duty or an internalized duty to act in ways that benefit the environment (López-Mosquera and Sánchez, 2012; Wyss et al., 2022). Han (2015a) discovered that when examining the intention of travelers to participate in an environmentally responsible convention, the ascription of responsibility has a beneficial impact on personal norms. Similarly, Setiawan (2021) conducted a study in China that discovered a strong positive relationship between the ascription of responsibility and personal norms in consumers to promote the adoption of electric vehicles using moral norms. In the present study, therefore, we anticipate that individuals who feel responsible for the consequences of harmful environmental actions are more inclined to form a moral obligation to behave pro-environmentally. To this end, the following hypothesis is proposed:

Hypothesis 5b: Ascription of responsibility positively and significantly relates to an individual’s personal norms.

#### 2.2.6 Awareness of consequences and pro-environmental behavior

Savari et al. (2023) state that individuals are more inclined to take actions that are beneficial to the environment when they understand how their behavior affects environmental concerns. The Norm Activation Model (NAM) is often employed to examine this relationship. It indicates that awareness of consequences (AC) plays a key role in shaping pro-environmental choices by fostering a sense of moral duty to engage in eco-friendly actions. De Groot and Steg (2009) proposed that individuals with a heightened awareness of the potential environmental outcomes of their actions, shaped by their exposure, dependence, and attention to media coverage of environmental issues, are likely to develop stronger personal values and principles. Consistent with findings by Zhang and Dong (2020), earlier research indicates that individuals aware of environmental consequences are inclined to endorse and purchase sustainably manufactured products with minimal environmental impact, motivating businesses to implement eco-friendly practices. Klöckner (2013) and Chen (2015) argued that when people believe their behavior has an environmental impact, they can become more willing to engage in pro-environmental behavior. Furthermore, other studies have also highlighted the effects of awareness on engaging in environmentally friendly actions across various contexts, such as participation in green electricity, vehicle ownership and usage, water recycling (Saphores et al., 2012), and land management (Price and Leviston, 2014). Based on these arguments, the following hypothesis is proposed.

Hypothesis 6: Awareness of environmental consequences positively affects individuals’ pro-environmental behavior.

#### 2.2.7 Ascription of responsibility and pro-environmental behavior

Having a clear understanding of the individuals or entities responsible for environmental problems can significantly influence the actions taken by individuals and groups. Individuals who ascribe responsibility to themselves for environmental issues are more inclined to endorse and demonstrate behaviors that benefit the environment (Han, 2015a). Previous research examined and found evidence of the impact of attributing responsibility in various areas, including green accommodation (Han, 2015a, 2015b), usage of renewable electricity, adoption of eco-innovation (Jansson et al., 2010), and waste reduction. Similarly, according to the norm activation theory, when individuals perceive themselves as responsible for environmental problems, they are more inclined to engage in pro-environmental actions (Schwartz, 1977). This feeling of individual responsibility can prompt individuals to take steps toward minimizing their impact on the environment. Hence, the following hypothesis is proposed.

Hypothesis 7: Ascription of responsibility positively affects individuals’ pro-environmental behavior.

#### 2.2.8 Personal norms and pro-environmental behavior

Awareness of consequences and ascription of responsibility can impact one’s actions towards the environment by stimulating personal norms to behave pro-environmentally. According to Ruepert et al. (2016), personal norms are an individual’s expectations and are perceived as a sense of moral responsibility to participate in the appropriate behavior. Individuals who possess a strong sense of personal norms towards the environment feel a moral obligation to act environmentally friendly (Van der Werff et al., 2013). Personal norms can vary in scope, ranging from broad expectations, such as promoting pro-environmental actions overall, to more specific ones, like adhering to recycling practices or turning off lights when they are not needed. Studies revealed that strong general, as well as specific environmental personal norms, indeed encourage many different pro-environmental behaviors, such as turning off the tap while brushing one’s teeth, willingness to pay higher prices for environmentally friendly food, intention to participate in actions to reduce emissions of particulate matters (Steg and De Groot, 2010), reductions in car use, as well as pro-environmental actions in general. Likewise, Ateş (2020), in his study aimed at investigating factors influencing pro-environmental behavior within the scope of the Theory of Planned Behavior and Value identity personal norm model, found that personal norms had a direct effect on pro-environmental behavior. In a similar vein, De Groot et al. (2021) discovered a robust and positive relationship between an individual’s personal norms and their pro-environmental actions such that the stronger an individual’s personal norm is regarding a specific pro-environmental behavior, the more likely they are to exhibit stronger behaviors associated with that behavior. In the NAT, personal norms play a crucial role, and it is believed that the impact of situational and personality trait activators occurs through personal norms. This means that personal norms mediate between these activators and their influence on behavior (Harland et al., 2007). Therefore, we proposed the following hypothesis.

Hypothesis 8: Personal norms positively affect pro-environmental behavior.

#### 2.2.9 The mediating effect of personal norms in the relationships among awareness of consequences, the ascription of responsibility, and pro-environmental behavior

When individuals become aware of the negative outcomes of not behaving in an environmentally friendly manner, their personal norms will guide their behavior. These personal norms, influenced by an individual’s sense of moral obligation, are initially activated and shape their actions (Setiawan, 2021). As a result, these personal norms promote the acceptance and practice of environmentally friendly actions (Mouro and Duarte, 2021). Some research conducted in environmental studies has discovered that activators can shape behavior by influencing personal norms. For instance, Zhang et al. (2013) and Chou (2014) demonstrated that engaging individuals’ norms through awareness of consequences and taking personal responsibility can lead to increased behaviors that support the environment. Similarly, Ateş (2020) discovered that personal norms mediated the relationship between environmental self-identity and pro-environmental behavior such that environmental self-identity had indirect influence on pro-environmental behaviors via personal norms. In this regard, the following hypothesis is proposed.

Hypothesis 9a: Personal norms will mediate the direct relationship between awareness of consequences and pro-environmental behavior.

Hypothesis 9b: Personal norms will mediate the direct relationship between ascription of responsibility and pro-environmental behavior.

#### 2.2.10 The moderating role of self-efficacy in the relationship between personal norms and PEB

**Figure 1:**
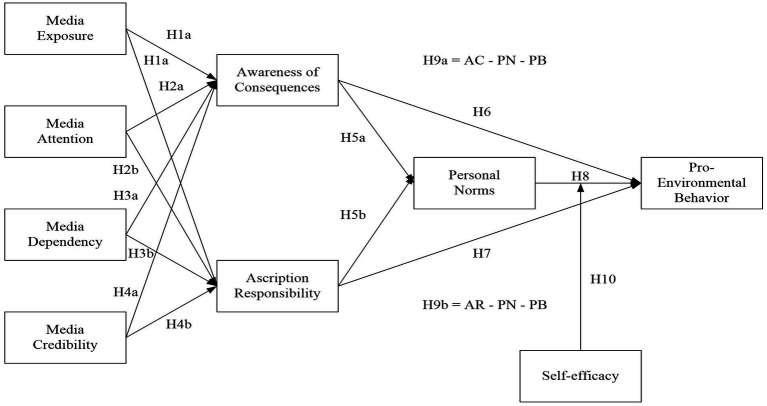
Research framework.

Self-efficacy, an individual’s confidence in achieving desired outcomes and influencing circumstances, plays a crucial role in emotions, thoughts, behaviors, and motivation for success (Gong et al., 2022; Panadero et al., 2017). However, environmentally friendly actions may require additional time, availability, and financial resources (Bouman et al., 2021). Acknowledging one’s ability to engage in pro-environmental behaviors is essential. Guided by the Norm Activation Theory (NAT), this study explores the impact of self-efficacy on the relationship between personal norms and Pro-Environmental Behaviors (PEB), investigating whether self-efficacy enhances or diminishes this connection. Previous research (Patrick et al., 2018) indicates that a strong belief in one’s abilities (self-efficacy) in social situations leads to confidence in engaging in behaviors that benefit others. The NAT suggests that personal norms are stronger when individuals can actively participate in actions to address environmental issues (Steg and Nordlund, 2018). Therefore, based on the above argument, the following hypothesis is proposed. Additionally, Figure 1 illustrates the research framework of SEM.

Hypothesis 10: The relationship between personal norms and pro-environmental behavior is moderated by the level of an individual’s self-efficacy, such that the relationship is stronger when self-efficacy is high and weaker when self-efficacy is low.

## 3 Research methods

The research uses a unique method, combining Fuzzy decision-making trial and evaluation laboratory (F-DEMATEL) and structural equation modeling (SEM) to examine cause-and-effect relationships and causal relationships between factors. Two separate studies were conducted, one using F-DEMATEL to investigate the influence of media factors on personal behaviors (awareness of consequences and ascription of responsibility) (study 1) and the other using SEM to validate theories and examine the relationship between media factors, awareness of consequences, ascription of responsibility, personal norms, self-efficacy, and PEB (study 2). This section provides an overview of the survey participants, explains the fuzzy DEMATEL approach, its objectives, and procedural structure, and explains the use of the cross-sectional approach and SEM analytical tool. It covers sample size determination, questionnaire design, measurement item selection, and data analysis methodology.

### 3.1 Data analytical tools

The current research employs two studies involving the use of two distinct methods. The study utilizes the F-DEMATEL approach and SEM as the primary analytical tools for examining the relationships among the constructs. According to Si et al. (2018), the DEMATEL method is a structured technique to analyse complex relationships and interdependencies among variables within a specified system. It provides a structured framework to assess the cause-and-effect relationships among various factors and components (Fang et al., 2021). This technique is employed to address complex relationships and has the potential to enhance comprehension of particular cluster issues by offering a hierarchical framework, thus offering practical solutions (Tzeng et al., 2005). The DEMATEL methodology can effectively analyze the interrelationships and structure of various factors when making complex decisions (Zhou et al., 2011). It has been proven useful in many areas, including marketing strategy, social marketing, environmental concerns, information systems, service quality, and the business ecosystem. Although DEMATEL plays a significant role in decision-making, it is unsuitable for addressing the complexities of uncertain situations when multiple factors are involved. This is because organizational problems often occur under uncertain conditions. Consequently, developing an expanded DEMATEL approach by utilizing fuzzy theory becomes essential.

Fuzzy theory refers to applying fuzzy mathematical techniques in addressing challenging decision-making issues (Aaldering et al., 2018). These problems comprise large systems with complicated relationships in which the variables cannot be accurately assigned (Liao, 2022). Given that numerous concepts in the real world lack precise definitions, the application of fuzzy decision-making theory proves to be a highly effective method for addressing these ambiguous and intricate issues.

#### 3.1.1 F-DEMATEL description

Fuzzy logic is essential due to the difficulty in assessing human preferences using precise numerical values. The F-DEMATEL method, a structural model based on fuzzy set theory, illustrates cause-and-effect relationships between criteria (Lin, 2013). Its primary advantage is its ability to consider fuzzy conditions and adapt to ambiguous situations, making it a valuable tool for understanding human preferences (Abdullah et al., 2022). The F-DEMATEL technique, based on linguistic variables and expert input, is used in this study to identify media factors influencing environmental awareness and responsibility ascription. This approach confirms cause-and-effect relationships among criteria, reduces uncertainty in subjective evaluation by experts, and increases representative reliability (Liao, 2020). The F-DEMATEL method involves the following computation steps (Lin, 2013; Liao, 2020; Abdullah et al., 2022).

##### 3.1.1.1 Step 1: select and collect the viewpoint of the experts on the research issue

The study sought to establish causal relationships between media factors and environmental awareness, along with the ascription of responsibility. A survey involving 20 communication and environmental experts was conducted to validate these cause-and-effect connections.

##### 3.1.1.2 Step 2: design and define the fuzzy linguistic scale

The fuzzy linguistic scale has been introduced to address the inherent vagueness in human assessments. This scale is utilized to transform direct impacts into triangular fuzzy numbers, as outlined in Table 1. The linguistic variable “influence” employs a five-level scale encompassing the following terms: no influence, very low influence, low influence, high influence, and very high influence. Table 1 illustrates the fuzzy numbers corresponding to different linguistic terms.

**Table 1:** The correspondence of linguistic terms and values.

Linguistic terms	Linguistic values
Very high influence	(0.75, 1.00, 1.00)
High influence	(0.50, 0.75, 1.00)
Low influence	(0.25, 0.50, 0.75)
Very low influence	(0.00, 0.25, 0.50)
No influence	(0.00, 0.00, 0.25)

##### 3.1.1.3 Step 3: compute the initial direct-relation fuzzy matrix Zk

The matrix Zk is constructed by having evaluators establish fuzzy pairwise influence relationships between components in an n×n matrix, where k represents the number of experts. Letting i=1 , 2,3, n denoted the n evaluation criteria, the *p* experts compare criteria in pairs to generate $Z^{\left(1\right)}$ , $Z^{\left(2\right)}$ , …, $Z^{\left(p\right)}$ . The fuzzy matrix $Z^{k}$ serves as the initial matrix of direct-relation fuzzy relationships for expert k, defined by Equation 1:


(1) $$Z^{k}=x_{\mathrm{ij}}^{k}$$

where **Z** is an *n* × *n* non-negative matrix, *x_ij_* reflects the direct influence of component *i* on factor *j*; and, when *i* = *j*, the diagonal elements *x_ij_* = 0, *k* = 1,2,3…*p*.

##### 3.1.1.4 Step 4: calculate the normalize direct-relation fuzzy matrix


(2) $$r^{k}=(\sum_{j=1}^{n}u_{\mathrm{ij}}^{k})\;k=1,2\ldots ,p$$

The linear scale transformation is then applied to compare the criteria, followed by obtaining the normalized direct-relation fuzzy matrix as $X^{\left(K\right)}$ .


(3) $$X^{\left(K\right)}=[\begin{array}{ccc} \begin{array}{cc} X_{11}^{\left(k\right)} & X_{12}^{\left(k\right)}\\ X_{21}^{\left(k\right)} & X_{22}^{\left(k\right)} \end{array} & \cdots & \begin{array}{c} X_{1n}^{\left(k\right)}\\ X_{2n}^{\left(k\right)} \end{array}\\ \vdots & \ddots & \vdots \\ X_{n1}^{\left(k\right)}\;\;X_{n2}^{\left(k\right)} & \cdots & X_{\mathrm{nn}}^{\left(k\right)} \end{array}]\;k=1,2\ldots ,p$$

Where


$$X_{\mathrm{ij}}^{\left(K\right)}=(L_{\mathrm{ij},}^{\left(k\right)}M_{\mathrm{ij},}^{\left(k\right)}U_{\mathrm{ij}}^{\left(k\right)})=(\frac{Z_{\mathrm{ij}}^{\left(k\right)}}{r^{\left(k\right)}})=(\frac{l_{\mathrm{ij}}^{\left(k\right)}}{r^{\left(k\right)}},\frac{m_{\mathrm{ij}}^{\left(k\right)}}{r^{\left(k\right)}},\frac{u_{\mathrm{ij}}^{\left(k\right)}}{r^{\left(k\right)}}).$$

The following Equation 4 is used to find the average matrix of superscript for *k* = 1, 2, …, *p.*


$$\;L=[\begin{array}{ccc} L_{11} & \cdots & L_{1n}\\ \vdots & \ddots & \vdots \\ L_{n1} & \cdots & L_{\mathrm{nn}} \end{array}],M=[\begin{array}{ccc} M_{11} & \cdots & M_{1n}\\ \vdots & \ddots & \vdots \\ M_{n1} & \cdots & M_{\mathrm{nn}} \end{array}],U=[\begin{array}{ccc} U_{11} & \cdots & U_{1n}\\ \vdots & \ddots & \vdots \\ U_{n1} & \cdots & U_{\mathrm{nn}} \end{array}]$$

where


(4) $$L_{\mathrm{ij}}=\frac{1}{p}\sum \begin{array}{c} p\\ k=1 \end{array}L_{\mathrm{ij}}^{\left(k\right)},M_{\mathrm{ij}}=\frac{1}{p}\sum \begin{array}{c} p\\ k=1 \end{array}M_{\mathrm{ij}}^{\left(k\right)},U_{\mathrm{ij}}=\frac{1}{p}\sum \begin{array}{c} p\\ k=1 \end{array}U_{\mathrm{ij}}^{\left(k\right)},$$

##### 3.1.1.5 Step 5: generate and analyse structure model

The total-relation fuzzy matrix T is obtained by normalizing the direct-relation fuzzy matrix. The element tij represents the indirect influence relationship between factors i and j . The matrix T reflects the overall impact relationships between elements. The calculation of the total-relation fuzzy matrix ( T ) is expressed by the following Equations 5–8:


$$T=\lim_{m\to \infty }(X+X^{2}+X^{3}\ldots +X^{m}\;)=X*{\left(1-X\right)}^{-1}$$

where


(5) $$\mathrm{TL}=[TL_{\mathrm{ij}}]=\lim_{m\to \infty }(L+L^{2}+L^{3}\ldots +L^{c}\;)=L*{\left(1-L\right)}^{-1}$$

(6) $$\mathrm{TM}=[TM_{\mathrm{ij}}]=\lim_{m\to \infty }(M+M^{2}+M^{3}\ldots +M^{c}\;)=M*{\left(1-M\right)}^{-1}$$

(7) $$\mathrm{TU}=[TU_{\mathrm{ij}}]=\lim_{m\to \infty }(U+U^{2}+U^{3}\ldots +U^{c}\;)=U*{\left(1-U\right)}^{-1}$$

(8) $$T=[\begin{array}{ccc} \begin{array}{ccc} TL_{11}, & TM_{11}, & TU_{11}\\ TL_{21}, & TM_{21}, & TU_{21} \end{array} & \cdots & \begin{array}{ccc} TL_{1n}, & TM_{1n}, & TU_{1n}\\ TL_{2n}, & TM_{2n}, & TU_{2n} \end{array}\\ \vdots & \ddots & \vdots \\ TL_{n1},\;TM_{n2},\;TU_{n3} & \cdots & \begin{array}{ccc} TL_{\mathrm{nn}}, & TM_{\mathrm{nn}}, & TU_{\mathrm{nn}} \end{array} \end{array}]$$

##### 3.1.1.6 Calculate the influence degree, affected degree, center degree, and cause degree of each factor

The degree of influence indicated as *D_i_,* measures the extent to which different factors have a cumulative effect on other factors. Thus,


(9) $$D_{i=\sum_{j=1}^{n}\mathrm{tij}}$$

The affected degree *R_j_* indicates the extent to which the other factors influence each factor. Thus,


(10) $$R_{j=\sum_{i=1}^{n}\mathrm{tij}}$$

The center degree, *R_j_*−*D_i_*, indicates the importance of factors. Therefore, the center degree:


(11) $$\left\{(R_{j}+D_{i})|i=j\right\}$$

For the cause degree, when $R_{j}+D_{i}$ is positive, the factor belongs to the cause group, and when $R_{j}-D_{i}$ is negative, the factor belongs to the effect group.


(12) $$\text{The cause degree}=\{(R_{j}-D_{i})|i=j\}$$

##### 3.1.1.7 Step 7: establish and analyse the F-DEMATEL diagram

**Figure 2:**
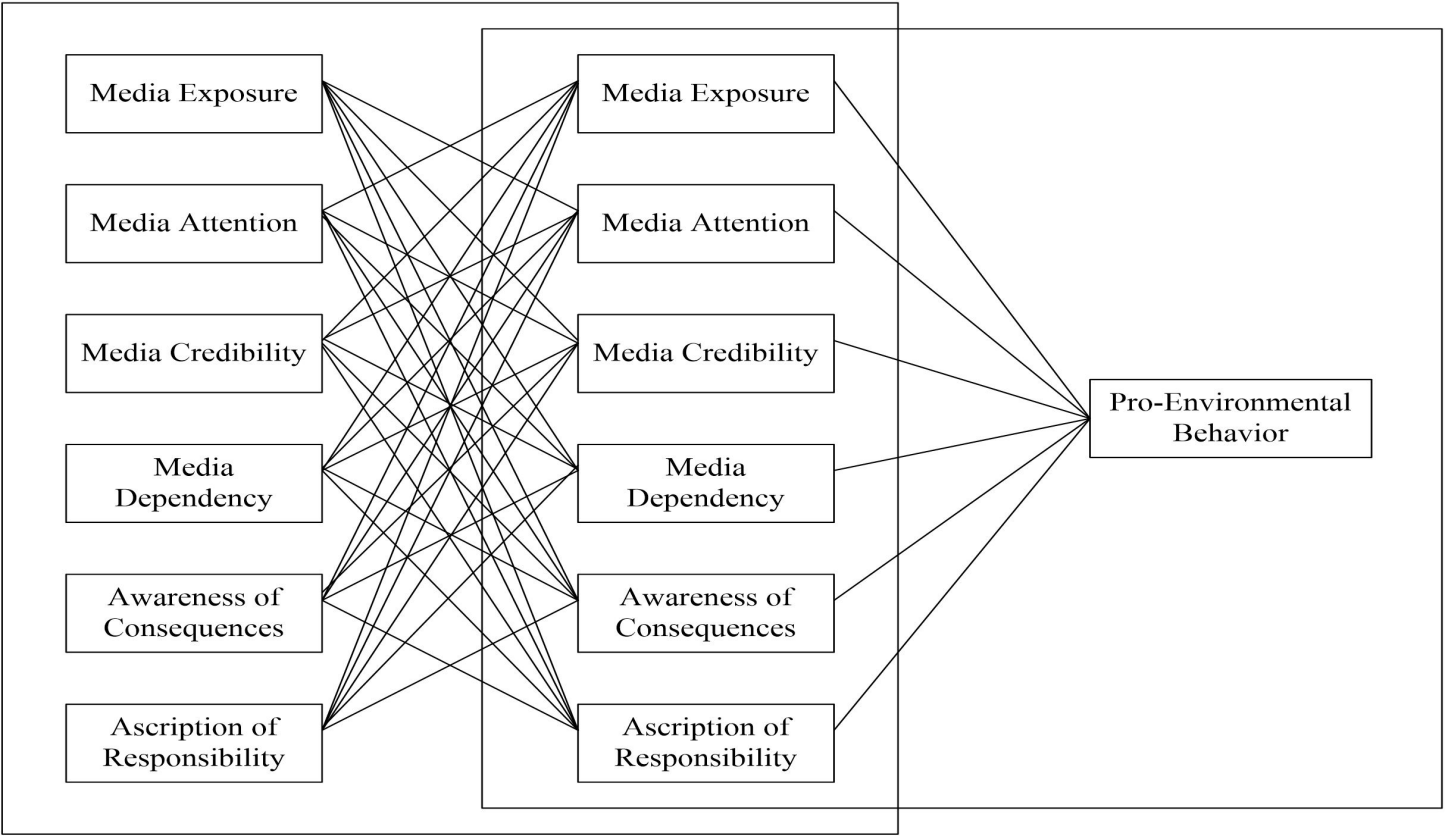
DEMATEL process framework.

In the total-relation matrix T the sum of rows and columns is represented independently by the vectors $R_{j}$ and $D_{i}$ . By mapping the dataset of ( $R_{j}+D_{i},R_{j}-D_{i}$ ), a cause-and-effect graph can be generated. The cause-and-effect graph is formed by mapping the dataset of ( $R_{j}+D_{i}$ ) on the horizontal axis, labeled as “Prominence,” indicating the criterion’s relevance. Simultaneously, the vertical axis ( $R_{j}-D_{i}$ ), labeled as “Relation,” is created by subtracting $D_{i}$ from $R_{j},$ aiding in organizing criteria into a cause category. Figure 2 illustrates the DEMATEL process framework.

#### 3.1.2 Structural equation modelling (SEM)

SEM is a data analysis method that employs regression analysis to predict outcomes by considering relationships among multiple variables. It integrates factor analysis and path analysis to validate empirical research and explore causal connections between variables (Lin et al., 2020). SEM involves constructing a theoretical model, identifying and estimating parameters through path diagrams, assessing the model’s fit, and making adjustments as necessary (Fan et al., 2020).

### 3.2 Research participants

#### 3.2.1 Fuzzy DEMATEL (media experts)

The current research used a targeted approach to select participants, specifically targeting individuals with significant knowledge and experience in the media and communication field. In particular, the study identified and recruited 20 managerial professionals with an average of 17.2 years of industry experience in these specific fields. The study gathered insights from industry experts to understand the relationship between media influences, consequences, and responsibility. The participants represented various professions, such as marketing, advertising, public relations, creative directors, communication, social media, digital media, and content management. Their expertise enhanced the analysis and ensured its reliability and significance. The study’s methodology was enhanced by including these experts, enhancing its understanding and relevance.

#### 3.2.2 SEM research participants

The study collected data from Taiwan’s general population, targeting adult individuals who receive environmental information through mass media. Online survey questionnaires were distributed via Google forms and social media platforms. Participation was optional, and consent was required. A back-translation method ensured questionnaire items were suitable for the local setting. The survey form was divided into two parts: a first part covering factors and a second part collecting demographic data. 1,900 individuals responded to the questionnaires and 1,594 responses were analyzed as the final data set after deleting data with missing values. Data with incomplete information results in various issues (Graham, 2009) for example, it may lead to a decrease in statistical power (the likelihood of the test rejecting the null hypothesis when it is incorrect. Incomplete data can also lead to biased parameter estimations (Kang, 2013), reduced representation of the samples and complexity to the study analysis which can results in invalid conclusions.

### 3.3 Measurement items

#### 3.3.1 Fuzzy DEMATEL

For fuzzy DEMATEL, a linguistic scale is used to judge the degree of influence between two factors by the experts. Five linguistic variables were used to represent the degree of influence between factors for all the criteria studied: VH = Very high influence, HI = High influence, LI = Low influence, VL = Very low influence, and NO = No influence (Liao, 2020).

#### 3.3.2 SEM questionnaire survey

The study utilized a structured multiple-item measurement approach derived from prior research on media exposure, attention, dependency, credibility, consequences awareness, responsibility ascription, personal norms, self-efficacy, and pro-environmental behavior. The scales were developed using 5-point rating scales with anchors, 1 = strongly disagree, 2 = disagree, 3 = neutral, 4 = agree, and 5 = strongly agree, except for media exposure, which had anchors like “never,” “rarely,” “sometimes,” “often,” and “always” and media attention with the following anchors; 1 = No attention at all, 2 = Little attention, 3 = Sometimes, 4 = Close attention, 5 = Very close attention. The research framework included nine constructs with a specific number of items associated with each, with some phrases modified to align with the study’s focus. ME was measured by asking respondents to indicate how often they see, read, and hear any environmental information in any of the following ways (Television, radio, print newspaper, or the internet) in the past 6 months (*α* = 0.786) (Lee and Cho, 2020; Ho et al., 2015). MA was judged by enquiring participants to indicate how much attention they pay to environmental news/messages from four types of media, i.e., television, print newspapers, radio, and the Internet, on a scale of 1 = no attention at all to 5 = very close attention (α = 0.794) (Gong et al., 2022). MD was also measured on a 5-point Likert scale (Gong et al., 2022). Sample items include “Reading newspapers, watching television, listening to radio or surfing the Internet helps me to find out about environmental issues” (α = 0.788). MC was measured by a 7-measurement scale item on a 5-point Likert scale (Calvo-Porral et al., 2014). Sample items include “Mass media provides reliable, trustworthiness and accurate information” and “Mass media is sincere and honest” (α = 0.781).

Participants assessed AC using a four-measurement scale item adapted from Macovei (2015) and Nguyen et al. (2015) on a 5-point Likert scale. Sample items read “I am aware of the serious health threat from poor environmental actions” and “I am aware of a serious environmental problem” (α = 0.824). Four items were used to measure AR on a 5-point Likert scale (Onwezen et al., 2013; Wang et al., 2019). Sample questions read “I feel responsible for taking care of the environment in my daily life” and “I feel responsible for the problems of environmental damage due to inappropriate actions (α = 0.774). For PN, four measurement items from Zhang et al. (2014) served as the measures on a 5-point Likert scale ranging from 1-strongly disagree to 5-strongly agree. Items include “I have the obligation to dissuade anyone from damaging the local environment” and “I have the obligation to comply with local environmental regulations and laws” (α = 0.788). Participants responded to four measurement items adapted from Huang (2016) to measure SE. Some of the items read, “I believe I have the ability to take action to mitigate environmental problems” and “I can solve environmental problems I meet” (α = 0.704). PEB was assessed by eight measurement items adapted from Mónus (2021) and Markowitz and Shariff (2012). Sample items include “I try to tell others that the environment is important” and “I try to save water by turning off the water when I brush my teeth” (α = 0.905).

The study assessed the demographic characteristics of participants, including gender, age, education, and employment status. Gender was determined by the number 1, representing males, and 2, representing females. Age was categorized into four groups: 18–25, 26–40, 41–55, and above 56. Education level was categorized as elementary school, high school, bachelor’s degree, or postgraduate. Employment status was determined by whether respondents were employed, self-employed, not employed, or students.

## 4 Research results

### 4.1 F-DEMATEL results

In the next step of the process, the initial direct-relation fuzzy matrix is computed. This matrix is constructed by having evaluators establish fuzzy pair-wise influence relationships between components in an n×n matrix, where n represents the number of criteria, and k is the number of experts. The resulting matrix is an essential element in the subsequent stages of the analysis. The process involves gathering input from experts to capture their perceptions of the influence relationships between various components. Table 2 provides a representation of this initial direct-relation fuzzy matrix.

**Table 2:** Direct relation fuzzy matrix.

Factors	ME	MA	MD	MC	AC	AR	ME	MA	MD	MC	AC	AR	ME	MA	MD	MC	AC	AR
ME	0.0000	0.4875	0.5625	0.5250	0.6125	0.7000	0.0000	0.7375	0.8125	0.7750	0.8625	0.9500	0.0000	0.9375	1.0000	0.9625	1.0000	1.0000
MA	0.4875	0.0000	0.4875	0.4500	0.6125	0.5250	0.7375	0.0000	0.7375	0.7000	0.8625	0.7750	0.9250	0.0000	0.9375	0.9375	1.0000	1.0000
MD	0.4500	0.4500	0.0000	0.5000	0.5875	0.6875	0.7000	0.7000	0.0000	0.7500	0.8375	0.9375	0.9250	0.9250	0.0000	0.9375	1.0000	1.0000
MC	0.5375	0.4875	0.5500	0.0000	0.6250	0.5500	0.7875	0.7375	0.8000	0.0000	0.8750	0.8000	0.9750	0.9375	0.9875	0.0000	1.0000	1.0000
AC	0.1375	0.2125	0.2250	0.2500	0.0000	0.5125	0.3875	0.4625	0.4750	0.5000	0.0000	0.7625	0.6375	0.7125	0.7250	0.7500	0.0000	0.9500
AR	0.2000	0.1000	0.2125	0.1000	0.3625	0.0000	0.4500	0.3500	0.4625	0.3500	0.6125	0.0000	0.7000	0.6000	0.7125	0.6000	0.8500	0.0000

ME, Media exposure; MA, Media attention; MD, Media dependence; MC, Media credibility; AC, Awareness of consequences; AR, Ascription of responsibility.

Following the computation of the average direct-relation fuzzy matrix, the next step involves calculating the Normalized Direct-Relation Fuzzy Matrix. This is achieved by applying a linear scale transformation to compare the criteria. The resulting matrix, denoted as cap X, is obtained through the application of Equations 2–4, as outlined in the methodology section. Table 3 presents the Normalized Direct-Relation Fuzzy Matrix.

**Table 3:** Normalized initial direct matrix (D).

Factors	ME	MA	MD	MC	AC	AR	ME	MA	MD	MC	AC	AR	ME	MA	MD	MC	AC	AR
ME	0.0000	0.1679	0.1902	0.1770	0.2079	0.2346	0.0000	0.1774	0.1931	0.1838	0.2055	0.2243	0.0000	0.1914	0.2021	0.1942	0.2021	0.2021
MA	0.1679	0.0000	0.1595	0.1505	0.2079	0.1770	0.1774	0.0000	0.1715	0.1651	0.2055	0.1838	0.1887	0.0000	0.1889	0.1889	0.2021	0.2021
MD	0.1505	0.1505	0.0000	0.1639	0.1990	0.2301	0.1651	0.1651	0.0000	0.1746	0.1993	0.2212	0.1862	0.1862	0.0000	0.1889	0.2021	0.2021
MC	0.1814	0.1637	0.1858	0.0000	0.2123	0.1858	0.1869	0.1745	0.1900	0.0000	0.2086	0.1900	0.1968	0.1889	0.1995	0.0000	0.2021	0.2021
AC	0.0401	0.0708	0.0752	0.0841	0.0000	0.1726	0.0875	0.1091	0.1122	0.1184	0.0000	0.1807	0.1252	0.1436	0.1463	0.1516	0.0000	0.1915
AR	0.0664	0.0353	0.0708	0.0353	0.1196	0.0000	0.1059	0.0840	0.1091	0.0840	0.1434	0.0000	0.1410	0.1223	0.1436	0.1223	0.1703	0.0000

ME, Media exposure; MA, Media attention; MD, Media dependence; MC, Media credibility; AC, Awareness of consequences; AR, Ascription of responsibility.

The structure model was analyzed by generating the total-relation fuzzy matrix T , derived from normalizing the direct-relation fuzzy matrix. The elements of T , denoted as tij , indicated the indirect influence relationship between factors i and j. Reflecting the overall impact relationship between elements, the influence matrix T was calculated using the formula *T* = $\lim_{m\to \infty }(X+X^{2}+X^{3}\ldots +X^{m}\;)$ = X* (1 $-X){}^{-1}$ , with an identical matrix involved. The results of the total-relation fuzzy matrix ( T ) can be found in Table 4.

**Table 4:** Total relation fuzzy matrix.

			T = *L(I-L)^1*						T = *M(I-M)^1*						T = *U(I-U)^1*			
Factors	ME	MA	MD	MC	AC	AR	ME	MA	MD	MC	AC	AR	ME	MA	MD	MC	AC	AR
ME	0.3039	0.4392	0.5006	0.4574	0.6421	0.6945	0.5720	0.7139	0.7717	0.7302	0.9134	0.9583	1.2462	1.3997	1.4687	1.4190	1.5976	1.6266
MA	0.4186	0.2682	0.4459	0.4097	0.5986	0.6046	0.6842	0.5259	0.7154	0.6787	0.8642	0.8773	1.3803	1.2146	1.4337	1.3903	1.5695	1.5980
MD	0.4072	0.3992	0.3098	0.4198	0.5950	0.6483	0.6810	0.6726	0.5748	0.6905	0.8670	0.9126	1.3725	1.3655	1.2685	1.3842	1.5626	1.5910
MC	0.4499	0.4297	0.4891	0.3005	0.6334	0.6450	0.7167	0.6997	0.7561	0.5627	0.8992	0.9160	1.4079	1.3951	1.4639	1.2535	1.5943	1.6233
AC	0.1794	0.1980	0.2234	0.2135	0.2134	0.3775	0.4498	0.4597	0.4925	0.4742	0.4774	0.6514	1.0930	1.1003	1.1510	1.1197	1.1252	1.3093
AR	0.1676	0.1411	0.1857	0.1460	0.2734	0.1813	0.4230	0.4020	0.4478	0.4091	0.5514	0.4452	1.0409	1.0222	1.0832	1.0351	1.1985	1.0747

ME, Media exposure; MA, Media attention; MD, Media dependence; MC, Media credibility; AC, Awareness of consequences; AR, Ascription of responsibility.

The next step involved calculating the influence degree, affected degree, center degree, and cause degree of each factor using the Equations 9–12 outlined in the methodology section. Sums of the rows ( $R_{j}$ ) and columns (*C_i_*) of the total relation fuzzy matrix ( T ) were computed to obtain the overall prominence and net effect. Tables 5, 6 present the sums of rows and columns and the prominence, net effects, and classification of criteria into causes and effects, respectively. Table 5 displays the sum of both rows and columns, while Table 6 presents the prominence, net effects, and classification of criteria into causes and effects.

**Table 5:** The sum of rows and columns.

			T = *L(I-L)^1*							T = *M(I-M)^1*							T = *U(I-U)^1*				
Factors	ME	MA	MD	MC	AC	AR	*R_i_*	ME	MA	MD	MC	AC	AR	*R_i_*	ME	MA	MD	MC	AC	AR	*R_i_*
ME	0.3039	0.4392	0.5006	0.4574	0.6421	0.6945	2.7338	0.5720	0.7139	0.7717	0.7302	0.9134	0.9583	4.6594	1.2462	1.3997	1.4687	1.4190	1.5976	1.6266	8.7578
MA	0.4186	0.2682	0.4459	0.4097	0.5986	0.6046	2.3271	0.6842	0.5259	0.7154	0.6787	0.8642	0.8773	4.3458	1.3803	1.2146	1.4337	1.3903	1.5695	1.5980	8.5864
MD	0.4072	0.3992	0.3098	0.4198	0.5950	0.6483	2.3721	0.6810	0.6726	0.5748	0.6905	0.8670	0.9126	4.3986	1.3725	1.3655	1.2685	1.3842	1.5626	1.5910	8.5443
MC	0.4499	0.4297	0.4891	0.3005	0.6334	0.6450	2.4977	0.7167	0.6997	0.7561	0.5627	0.8992	0.9160	4.5505	1.4079	1.3951	1.4639	1.2535	1.5943	1.6233	8.7380
AC	0.1794	0.1980	0.2234	0.2135	0.2134	0.3775	1.2258	0.4498	0.4597	0.4925	0.4742	0.4774	0.6514	3.0051	1.0930	1.1003	1.1510	1.1197	1.1252	1.3093	6.8986
AR	0.1676	0.1411	0.1857	0.1460	0.2734	0.1813	0.9276	0.4230	0.4020	0.4478	0.4091	0.5514	0.4452	2.6784	1.0409	1.0222	1.0832	1.0351	1.1985	1.0747	6.4546
*C_i_*	1.9266	1.8754	2.1545	1.9469	2.9559	3.1514		3.5267	3.4738	3.7583	3.5454	4.5727	4.7608		7.5408	7.4975	7.8691	7.6018	8.6478	8.8227	

ME, Media exposure; MA, Media attention; MD, Media dependence; MC, Media credibility; AC, Awareness of consequences; AR, Ascription of responsibility.

**Table 6:** The prominence, net effects, and classification of the criteria into causes and effects.

	*R_j_* + *D_i_*	*R_j_* - *D_i_*	Identity
ME	9.7150	1.0523	Cause
MA	9.3687	0.8042	Cause
MD	9.6990	0.5110	Cause
MC	9.6268	0.8974	Cause
AC	9.1019	−1.6823	Effect
AR	8.9318	−2.2247	Effect

ME, Media exposure; MA, Media attention; MD, Media dependence; MC, Media credibility; AC, Awareness of consequences; AR, Ascription of responsibility.

The values of ( $R_{j}-D_{i}$ ) determine the net effects or causes of the various components. A negative value of ( $R_{j}-D_{i}$ ) suggests that the factor is an effect factor. In contrast, a positive value indicates that the factor influences other factors. From the table above, the fuzzy DEMATEL results have demonstrated that ME, MA, MD, and MC are cause factors, while AC and AR are effect factors with negative values of ( $R_{j}-D_{i}$ ). The combined values $R_{j}+D_{i}$ provide insight into the overall prominence of the cause-and-effect index. From Table 6, it is evident that ME holds the highest prominence with an $R_{j}+D_{i}$ value of 9.7150 within the cause group, followed by MD, MC, MA in that order with *R_j_* + *D_i_* values of 9.6990, 9.6268, and 9.3687, respectively. In this regard, ME is the most influential determinant of AC and AR, among other cause factors in environmental communication, and that is, it plays a crucial role in shaping PEB among individuals.

**Figure 3:**
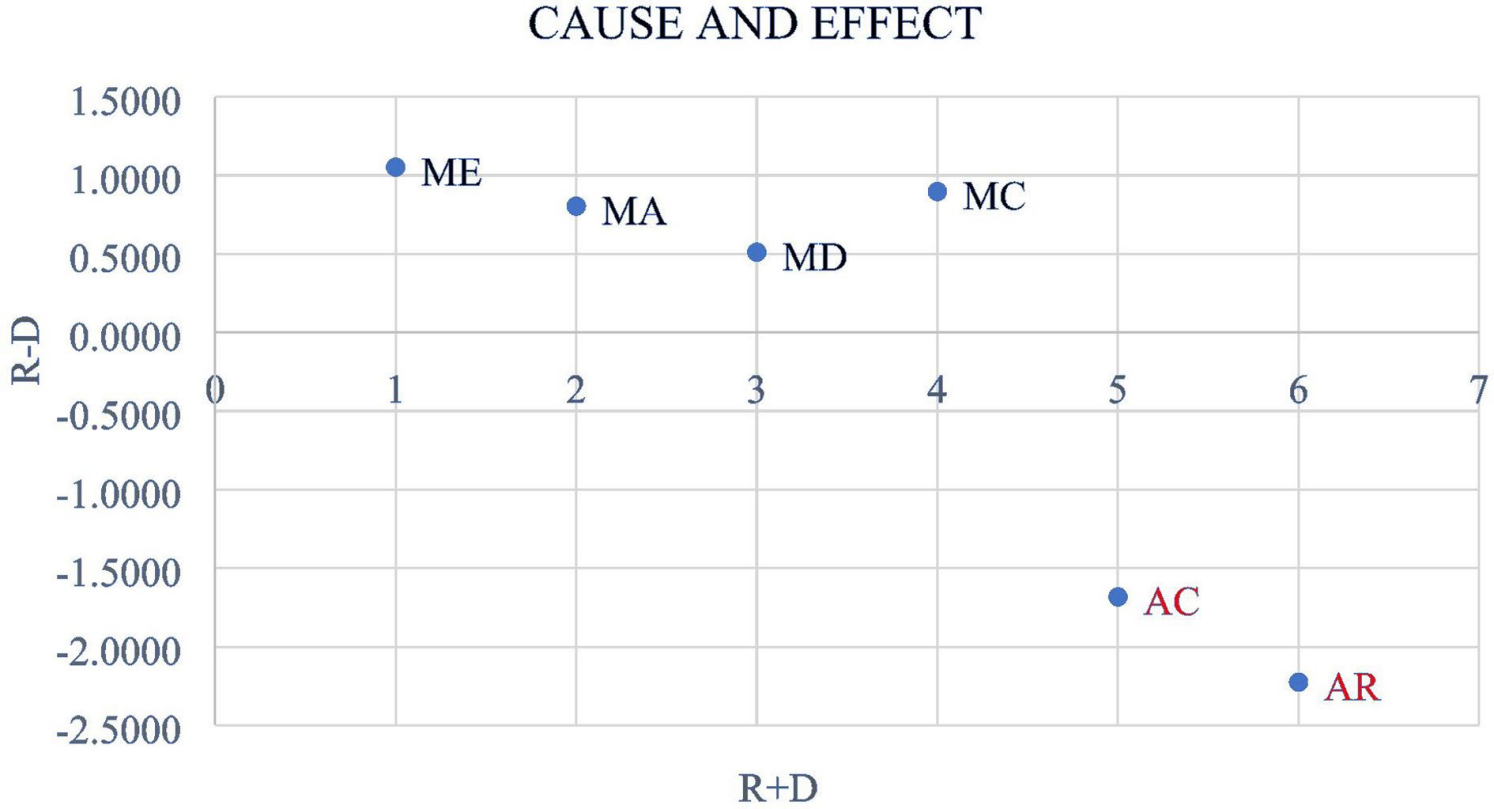
The cause-and-effect digraph. ME, Media exposure; MA, Media attention; MC, Media credibility; MD, Media dependency; AC, Awareness of consequences; AR, Ascription of responsibility.

Criteria in the effect group, those that the cause group influences, AC can be considered important as it has greater prominence with an $R_{j}+D_{i}$ value of 9.1019 above AR which has a value of 8.9318 with $R_{j}-D_{i}$ values of −1.6823 and −2.2247, respectively. The negative values of $R_{j}-D_{i}$ among criteria in the effect group signify that the cause group impacts them. In the final step, the F-DEMATEL cause-and-effect digraph is established and analyzed by mapping the dataset of $R_{j}+D_{i}\;$ and $R_{j}-D_{i}$ where the horizontal axis vector ( $R_{j}+D_{i}$ ), which is labeled as “Prominence,” was created by adding *R_j_* to *D_i_*, indicating the criterion’s relevance. In contrast, the vertical axis ( $R_{j}-D_{i})$ , labeled “Relation,” was created by subtracting *D_i_* from *R_j_*, which can be used to organize criteria into a cause category. Figure 3 depicts a cause-and-effect diagram visually representing the relationships among the factors and their causal influences.

### 4.2 SEM results

#### 4.2.1 Descriptive statistics of sample structure

The descriptive statistics of the sample’s structure are presented, highlighting various demographic characteristics. The demographic variables that also served as control variables included gender, age, employment status and educational qualifications. Out of the final 1,594 valid responses, 727 were males and 867 were females, making up 45.6 and 54.4% of the total, respectively with females being the majority. Participants indicated their age by circling the appropriate range and the age group 26–40 accounted for the highest participation with 677 individuals, making up 42.5% of the total. Regarding education, 51.6% (822 individuals) of the total valid sample held a bachelor’s degree, indicating the highest category while also the majority of the participants (46.2%) were employed.

#### 4.2.2 Multi collinearity assessment

The study used the Tolerance and Variance Inflation Factor (VIF) test to identify multicollinearity issues. The results showed that all constructs had VIF values below 10, indicating no multi-collinearity problems (James et al., 2013), with the highest VIF value being 1.802.

#### 4.2.3 Common method bias (CMB)

The study used Harman’s single factor test to check for common method bias (CMB). 43 factors were identified, accounting for 13.176% of the explained variance for the first factor. This suggests that the issue of CMB is not significant, as the first factor only accounts for less than 50% of the explained variance (Fuller et al., 2016).

#### 4.2.4 Measurement model assessment

##### 4.2.4.1 Composite reliability, discriminant, and convergent validity

Hair et al. (2011) recommends a reliability coefficient of 0.7 or higher for satisfactory reliability, and all nine variables in the study meet this requirement: ME = 0.72, MA = 0.70, MD = 0.75, MC = 0.91, AC = 0.75, AR = 0.77, PN = 0.72, SE = 0.70, and PEB = 0.91. The Composite Reliability (CR) of 0.7 ensures the reliability of all constructs (Hair et al., 2014). Average Variance Extracted (AVE) values, ranging from 0.55 to 0.70, mostly exceed the threshold of 0.5, which is considered satisfactory in many fields (Gefen and Straub, 2005). The CR values support reliability, and discriminant validity is maintained, with diagonal values greater than any values in a row or column corresponding to it. Table 7 presents validity and reliability values.

**Table 7:** Means, standard deviations, reliability coefficients, and correlations.

Variables	Mean	SD	α	CR	AVE	ME	MA	MD	MC	AC	AR	PN	SE	PEB
ME	3.17	0.95	0.79	**0.86**	**0.61**	0.78								
MA	3.39	0.99	0.79	**0.84**	**0.58**	0.55	0.76							
MD	4.31	0.60	0.79	**0.82**	**0.55**	0.17	0.29	0.74						
MC	4.34	0.41	0.78	**0.82**	**0.41**	0.13	0.31	0.22	0.64					
AC	4.27	0.65	0.82	**0.88**	**0.66**	0.10	0.16	0.08	0.21	0.81				
AR	4.43	0.51	0.77	**0.86**	**0.70**	0.20	0.23	−0.06	−0.04	−0.03	0.77			
PN	4.38	0.58	0.79	**0.86**	**0.61**	0.14	−0.03	0.08	0.01	0.25	0.37	0.78		
SE	4.25	0.65	0.70	**0.82**	**0.60**	−0.24	−0.37	−0.06	0.01	−0.09	−0.07	0.02	**0.74**	
PEB	4.24	0.67	0.91	**0.93**	**0.64**	0.11	0.051	0.13	0.14	0.20	0.10	0.25	−0.01	0.80

*N* = 1,594; *p < 0.05; **p < 0.01; ***p < 0.001. Reliability coefficients are reported along the diagonal parentheses where ME, Media exposure; MA, Media attention; MD, Media dependence; MC, Media credibility; AC, Awareness of consequences; AR, Ascription of responsibility; PN, Personal norms; SE, Self-efficacy; PEB, Pro-environmental behavior. The bold value represents the square roots of the Average Variance Extracted (AVE).

##### 4.2.4.2 Confirmatory factor analysis (CFA)

A Confirmatory Factor Analysis (CFA) was conducted in Covariance Based Structural Equation Modelling (CB-SEM) by using AMOS 22.0 to check for the goodness of fit of this study’s model (9 factor). A CFI value of greater than 0.90 is recognized as indicative of good fit (Hu and Bentler, 1999). In terms of GFI, values range between 0 and 1 and it is generally accepted that values of 0.90 or greater indicate well-fitting models (Hooper et al., 2008). For RMSEA fit, a value that is less than or equal to 0.06 indicates an adequate fit (Hu and Bentler, 1999) although the recommended lower limit is close to 0 and the upper limit should be less than 0.08 (MacCallum et al., 1996). Thus, Hair et al. (2011) recommended that the RMSEA within the range of 0.03–0.08 with 95% confidence interval need to be reported. Therefore, regarding the 9-factor model of the present study, the results indicated a good fit for the model, with a χ^2^ value of 161.877 and 12 degrees of freedom, and a reasonable level of fitness, as indicated by CFI values of 0.911, IFI of 0.912, GFI of 0.979, and RMSEA of 0.08 meeting the thresholds for model fit evaluation.

#### 4.2.5 Examination of research hypotheses

The study employed SEM to examine research hypotheses using AMOS 22. The test results indicated the magnitude and relevance of linkages between variables, either confirming or rejecting hypotheses. Path coefficients were evaluated on a scale, with values closer to +1 indicating stronger positive correlations while values closer to −1 indicating stronger negative correlations.

The results in Table 8 indicate that all the relationships except (AR to PN and AC to PEB) were supported as their *p*-values were below 0.05.

**Table 8:** Results of examination of direct research hypotheses.

Variables	Path coefficients	*P* Values	Remarks
ME → AC	0.132	***	Supported
MA → AC	0.048	0.023	Supported
ME→AR	0.169	***	Supported
MA → AR	0.043	0.036	Supported
MD → AC	0.139	***	Supported
MD → AR	0.106	***	Supported
MC → AC	0.419	***	Supported
MC → AR	0.095	0.002	Supported
AC → PN	0.081	***	Supported
AR → PN	0.021	0.355	Not Supported
AR → PEB	0.110	***	Supported
AC → PEB	−0.061	0.052	Not Supported
PN → PEB	0.530	***	Supported
Model fitness: χ^2^ = 161.877, df = 12, χ^2^/df = 13.4898, RMSEA = 0.08, CFI = 0.911, IFI = 0.912, GFI = 0.979			

ME, Media exposure; MA, Media attention; MD, Media dependence; MC, Media credibility; AC, Awareness of consequences; AR, Ascription of responsibility; PN, Personal norms; PEB, Pro-environmental behavior. ***p < 0.001.

The results presented in Table 8 indicate significant and positive relationships supporting several hypotheses. Specifically, ME was significantly and positively related to AC (*β* = 0.132, *p*-value<0.001), supporting hypothesis 1a. Similarly, ME was found to be significantly and positively associated with AR as proposed (β = 0.169, *p*-value<0.001), supporting hypothesis 1b. Hypothesis 2a, suggesting a significant and positive relationship between MA and AC, is supported with a coefficient of 0.048 and a *p*-value of 0.023. Additionally, hypothesis 2b, positing a significant and positive relationship between MA and AR, is supported with a coefficient of 0.043 and a *p*-value of 0.036. Hypotheses 3a and 3b, suggesting a significant relationship between MD and AC and AR, are supported with *p*-values below 0.001 and coefficients of 0.139 and 0.106, respectively. Finally, hypotheses 4a and 4b, indicating the influence of MC on AC and AR, are well supported with *p*-values below 0.001 and 0.002 and coefficients of 0.419 and 0.095, respectively.

**Figure 4:**
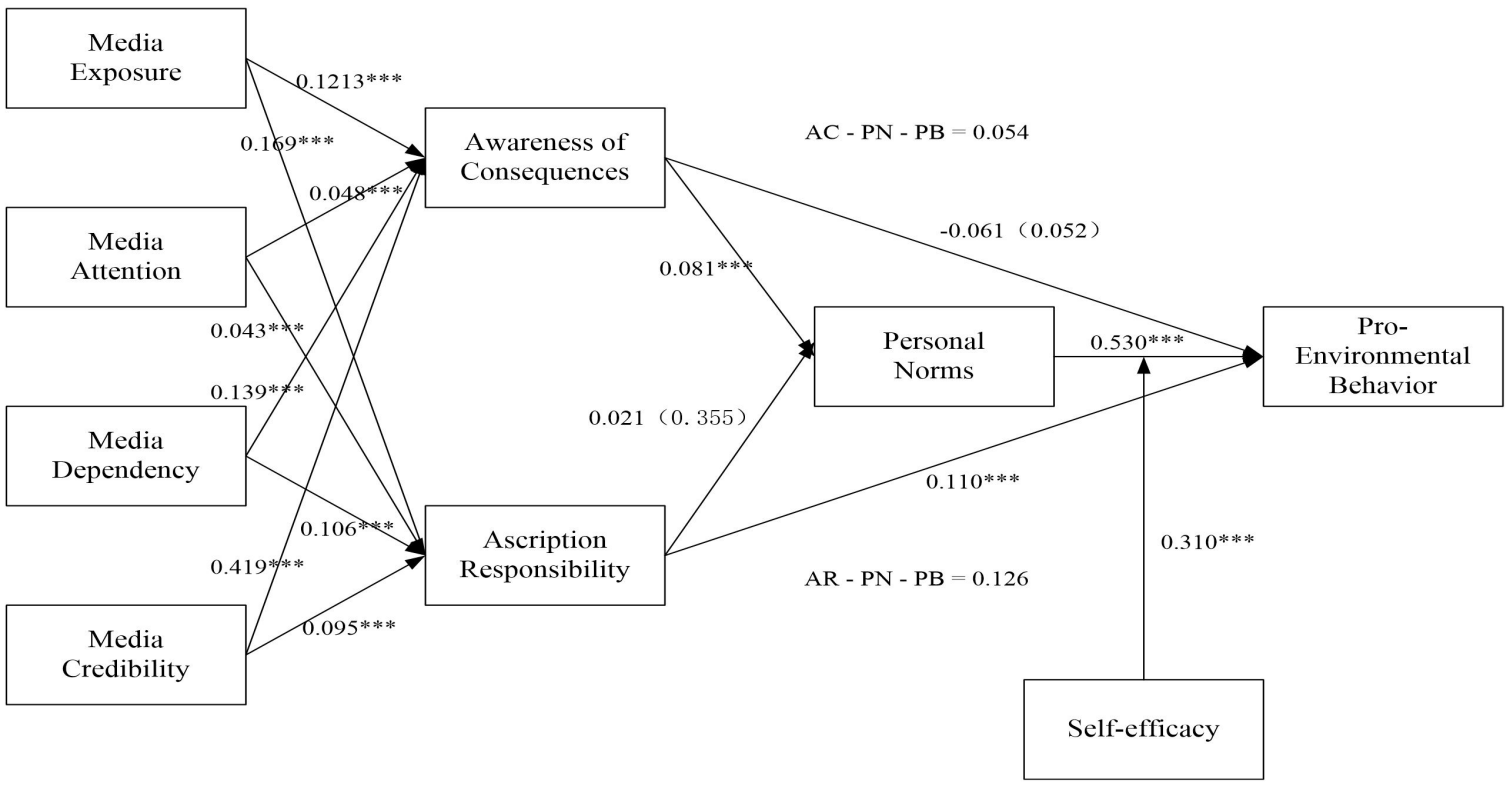
Path estimates for SEM.

The study’s findings align with several hypotheses. AC is significantly and positively related to PN (β = 0.081, *p*-value<0.001), supporting hypothesis 5a. However, for hypothesis 5b, which proposed a significant and positive relationship between AR and PN, the results did not support the hypothesis, indicated by the insignificant *p*-value of 0.355. Regarding the direct relationship between AC and PEB, the study found no significant relationship, with a *p*-value of 0.053 and a negative coefficient of −0.061, suggesting that AC does not directly impact PEB. Therefore, hypothesis 6 is not supported. On the other hand, there is a direct and significant relationship between AR and PEB, supported by a *p*-value below 0.001 and a coefficient of 0.110, validating hypothesis 7. Finally, the study establishes a robust, significant, and positive relationship between PN and PEB, with a *p*-value below 0.001 and a coefficient of 0.530, supporting hypothesis 8. The Path Estimates for SEM are depicted in Figure 4.

##### 4.2.5.1 Mediation effect analysis

A Bootstrap analysis was performed in SPSS using PROCESS macro 4.1 (Model 4) to determine the mediation effects. A non-zero value within the 95% confidence interval suggests that the mediation effect is significant and, therefore, exists (Cheung and Lau, 2011). The results of bootstrapping for indirect effects and mediation analysis are presented in Tables 9, 10.

**Table 9:** Bootstrapping results for indirect effects.

Rival Path	Estimated effect	Bootstrapping 95% CI
		Bias-corrected (LL, UL)
AC → PN → PEB	0.054	(0.031, 0.080)
AR → PN → PEB	0.126	(0.019, 0.163)

AC, Awareness of consequences; AR, Ascription of responsibility; PN, Personal norms, PEB, Pro-environmental behavior.

**Table 10:** Results of mediation analysis.

Rival path	Estimated effect	*P* Values	Remarks
AC → PN → PEB	0.054	***	H9a-Supported
AR → PN → PEB	0.126	***	H9b-Supported

*N* = 1,296; ^*^p < 0.05; ^**^p < 0.01; ^***^p < 0.001; AC, Awareness of consequences; AR, Ascription of responsibility; PN, Personal norms, PEB, Pro-environmental behavior.

The findings show that the rival path (AC-PN-PEB) has a significant mediation effect as its 95% confidence interval does not contain zero (estimate = 0.054, 95% CI [(0.031, 0.080)]), supporting hypothesis 9a, which suggests that personal norms mediate the relationship between awareness of consequences and PEB. The rival path (AR-PN-PEB) also has a significant mediation effect (estimate = 0.126, 95% CI [0.019, 0.163]), supporting hypothesis 9b, indicating that personal norms mediate the relationship between ascription of responsibility and PEB. This suggests that AC and AR indirectly influence PEB through personal norms.

##### 4.2.5.2 Moderation analysis

**Figure 5:**
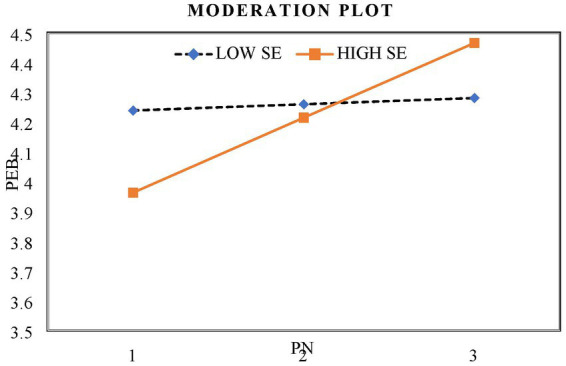
The moderating influence of self-efficacy on the relationship between personal norms and pro-environmental behavior. PN, Personal norms; SE, Self-efficacy; PEB, Pro-environmental behavior.

The moderation test conducted to examine hypothesis 10, proposing the interaction between personal norms and self-efficacy influencing PEB, yielded positive and significant results (b = 0.31, *p* < 0.001). This finding indicates that the impact of personal norms on PEB is moderated by self-efficacy. Participants with higher-than-average self-efficacy levels exhibited a stronger influence of personal norms on PEB compared to those with average or lower-than-average self-efficacy levels. The relationship between personal norms and PEB varies across different levels of self-efficacy, being more pronounced when self-efficacy is high and weaker when it is low. Consequently, hypothesis 10 is supported. The moderation graph illustrating the relationship between personal norms and PEB under high and low levels of self-efficacy is presented in Figure 5.

## 5 Discussion and conclusion

The study employs MSDT and NAT to investigate media’s influence on pro-environmental behaviors in the private sphere, aiming to fill research gaps regarding mass media’s impact on individual behavior, awareness of consequences, responsibility attribution, and the formation of personal norms affecting PEB. Predicting PEB through F-DEMATEL and SEM, the study identifies communication factors triggering NAT components. It examines the indirect effects of media factors on PEB through NAT’s personality trait elements using MSDT. Additionally, the study explores how self-efficacy moderates the relationship between personal norms and PEB.

The F-DEMATEL findings reveal that media exposure, attention, dependency, and credibility significantly influence awareness of consequences and responsibility regarding environmental concerns. Mass media is crucial in raising public awareness and attention to environmental issues and fostering understanding, responsibility, and engagement in environmental activities. Consistent with the F-DEMATEL results in study 1, the findings from the SEM-AMOS 22 also demonstrated positive and significant relationships among media factors and awareness of consequences as well as ascription of responsibility. The results revealed that factors in the NAT, which include understanding of consequences and ascription of responsibility, were positively and significantly influenced by those elements in the MSDT, which include media exposure, media attention, media dependency, and media credibility. Thus, remarkably consistent with the study’s prediction, media exposure was positively related to awareness of consequences and ascription of responsibility (Hypotheses 1a and b). This result accords with Ando et al. (2021), who suggested that being exposed to mass media or participating in mass media campaigns can effectively affect individuals’ awareness of consequences as they increase their concern for and knowledge about important environmental issues. The results are also in line with Wang et al. (2022), who stated that the use of mass media campaigns is a standard method to address environmental concerns by bringing attention to the public and instilling a sense of responsibility towards these issues and that these campaigns aim to increase awareness of environmental problems and promote a collective commitment towards their resolution. Therefore, someone who is aware that their actions have negative impacts on others and the environment may feel responsible for these effects.

The positive relationships between media attention and awareness of consequences as well as ascription of responsibility imply that the more individuals pay attention to mass media on issues concerning the environment, the more they get to know the consequences associated with poor environmental practices as well as feeling responsible for some environmental effects. Following these findings, Ho et al. (2015) argued that individuals who show a higher level of interest in environmental news tend to engage in extensive processing of the information and, hence, gain a greater understanding of the issue. Similarly, Karimi et al. (2021) suggested that environmental news on traditional media channels such as TV, radio, and newspapers has a vital impact on raising environmental awareness and understanding within different communities. Furthermore, the outcome that media dependency positively relates to awareness of consequences and ascription of responsibility accords with the media system dependency theory, which proposes that people rely more on mass media for information when they encounter uncertainty and societal disruptions, such as natural catastrophes (Kim, 2020). In this regard, individuals depend on mass media channels for information concerning the environment, hence being crucial in environmental communication. Individuals become more dependent on the mass media if their goals are being satisfied.

Media credibility positively relates to awareness of consequences and responsibility attribution. Trust in media sources is associated with taking environmental consequences seriously, as observed by Lee and Cho (2020). The study establishes a positive relationship between awareness of consequences and personal norms, consistent with NAT which emphasizes the importance of awareness of consequences as one of the factors activating personal norms (He and Zhan, 2018). The theory states that personal norms are triggered when individuals become aware of possible consequences and take responsibility for their actions (Setiawan, 2021). The result is also consistent with Liu et al. (2017), who contended that when people perceive a problem caused by a particular behavior, they act on that perception and consider how and whether it will help them solve the problem. However, contrary to the predictions of this study, the ascription of responsibility was not related to personal norms. This finding is inconsistent with the NAT, which states that a sense of responsibility activates people’s norms and leads them to engage in prosocial behaviors such as environmentally friendly behavior (Schwartz, 1977).

The non-significant relationship observed may be attributed to the possibility that individuals become aware of the consequences of their actions before assuming personal responsibility for them (De Groot and Steg, 2009). This suggests that the ascription of responsibility is likely shaped by awareness of consequences, which in turn precedes the activation of personal norms, thereby explaining the directional link between AC and AR. In addition, social desirability bias may have influenced the responses. Participants may have been reluctant to acknowledge or attribute socially undesirable behaviors to themselves, particularly when such behaviors contradict prevailing social norms (Kwak et al., 2019). This bias can undermine the accuracy of self-reported personal norms, potentially contributing to the lack of a significant association. Cultural context also plays a crucial role. Taiwanese society, which is rooted in collectivist cultural values, often emphasizes shared responsibility among group members or institutional actors over individual accountability (Yeh and Hsieh, 2025). Such a cultural orientation may dilute the impact of perceived personal responsibility on the formation of moral norms. Furthermore, cultural differences in the interpretation of abstract constructs, such as “responsibility” and “norms,” may diverge from Western conceptualizations, possibly affecting the cross-cultural validity of survey instruments. The demographic composition of the sample may also be a contributing factor. Younger participants, who comprised a portion of the sample, may have a less developed sense of moral responsibility regarding complex societal challenges, including environmental and social sustainability.

Results demonstrated an insignificant relationship between AC and PEB. This implies that awareness of consequences and pro-environmental behaviors were not related in the current study; hence, hypothesis 6 was not supported. This outcome contradicts the assertions of Zhang and Dong (2020) and Price and Leviston (2014), who contended that individuals cognizant of environmental consequences are inclined to endorse and buy sustainably produced products with minimal environmental impact. The insignificant relationship could be attributed to the tools and techniques utilized to gauge the relationship between the awareness of consequences, and pro-environmental behavior might not be sufficiently accurate or sensitive. As a result, they might have failed to identify any meaningful correlations that could exist. On the other hand, in line with our prediction, results revealed a positive relationship between ascription of responsibility and pro-environmental behavior. The finding accords with previous research, such as Han’s (2015a, 2015b) study, which proposed that individuals responsible for environmental issues are more likely to support and engage in eco-friendly behaviors.

Results also revealed a positive relationship between personal norms and pro-environmental behavior, consistent with our prediction rendering empirical support to hypothesis 8. The finding is in line with Van der Werff et al. (2013) and Ateş (2020) studies, which revealed that strong general as well as specific environmental personal norms encourage various pro-environmental behaviors, such as turning off the tap while brushing one’s teeth, reductions in car use, as well as pro-environmental actions in general. Furthermore, De Groot et al. (2021) discovered a robust and positive relationship between an individual’s personal norms and their pro-environmental actions such that the stronger an individual’s personal norm is regarding a specific pro-environmental behavior, the more likely they are to exhibit stronger behaviors associated with that behavior. Likewise, personal norms are crucial in the NAT as the impact of situational and personality trait activators occurs through it.

Under the mediation analysis, the study results confirm the mediating role of personal norms in the relationships between AC and PEB as well as AR and PEB, supporting hypotheses 9a and b. The findings show the existence of the mediation effect. They are in line with Setiawan (2021), Mouro and Duarte (2021), and Chou (2014), who argued that the activation of personal norms by awareness of consequences and ascription of responsibility would guide and shape individual’s behavior towards the environment. Personal norms promote the acceptance and practice of environmentally friendly actions. This finding strongly supports the application of NAT in environmental protection, as it demonstrates that personal norms play a crucial role in influencing behaviors that benefit the environment.

Consistent with the study’s prediction, self-efficacy demonstrated a positive moderating role in the relationship between personal norms and pro-environmental behavior, providing support to hypothesis 10. In this regard, high self-efficacy among individuals strengthens the relationship, and low self-efficacy weakens the personal norms-pro-environmental behavior relationship. The finding accords with prior research, such as Patrick et al. (2018), which have demonstrated that having a strong belief in one’s abilities (self-efficacy) in social situations leads to individuals feeling confident in engaging in behaviors that benefit others as well as lowering the likelihood of individuals disengaging from moral responsibilities. The finding is also in line with the NAT, which states that personal norms are stronger when people can engage in the actions needed to reduce environmental problems (Steg and Nordlund, 2018). According to NAT, personal norms are activated when individuals recognize an issue, such as environmental degradation, as personally significant and feel a corresponding sense of moral obligation to take action (Schwartz, 1977). However, the extent to which these norms translate into behavior is contingent upon an individual’s perceived self-efficacy. Within the context of PEB, self-efficacy functions as a pivotal mediator that enables individuals to convert moral intention into action. Those with high self-efficacy, who believe they can contribute meaningfully to environmental solutions, are more inclined to align their behaviors with their moral beliefs. Conversely, individuals with low self-efficacy may be less likely to engage in PEB, even when they endorse strong personal norms, due to skepticism regarding their capacity to produce meaningful change. Therefore, empirically, the study has demonstrated that self-efficacy profoundly impacts the personal norms-pro-environmental behavior relationship through its moderating role.

This study provides valuable insights into the interplay of media, personal norms, and self-efficacy in shaping pro-environmental behaviors, contributing to understanding effective environmental communication strategies.

## 6 Implications, limitations, and future research

### 6.1 Theoretical implications

This research holds significance in both theoretical and practical realms. The study sheds light on the pivotal role of mass media in environmental conservation, particularly in influencing individuals’ pro-environmental actions. By incorporating the Norm Activation Theory (NAT) and Media System Dependency Theory (MSDT), the study extends the understanding of media’s impact on activating components of the NAT, such as awareness of consequences, ascription of responsibility, and personal norms. Integrating media exposure, attention, credibility, and dependency as triggers for NAT components provides a nuanced understanding of how media contributes to shaping and guiding individuals’ pro-environmental behaviors (PEB). Additionally, this study is pioneering in exploring the moderating role of self-efficacy within the NAT framework, offering new insights into the interplay between personal norms and PEB. The findings suggest that self-efficacy enhances the influence of personal norms, emphasizing the importance of individuals’ belief in their ability to engage in actions addressing environmental issues. Overall, this research contributes valuable knowledge to environmental psychology and communication theories, offering practical implications for media campaigns promoting pro-environmental behaviors.

### 6.2 Practical implications

Understanding the connection between media elements, awareness of consequences, and the ascription of responsibility is vital for stakeholders like media corporations, decision-makers, and educators. Initiating media literacy programs to enhance analytical thinking in the face of diverse media forms is crucial. Public education should stress the importance of critically assessing information, grasping potential outcomes, accurately assigning responsibility, and fostering better public knowledge and accountability for environmental protection. With media factors influencing pro-environmental behaviors through components of the Norm Activation Theory (NAT), regular campaigns by media organizations can raise awareness and provide guidelines for environmentally friendly actions.

Promoting diverse media consumption is essential for increased dependence on and attention to media, cultivating a more inclusive worldview. Media organizations should employ various formats like videos, infographics, articles, and podcasts to cater to diverse preferences. Ensuring that environmental information is easily accessible, credible, and understandable is crucial to avoid hindering the development of personal norms and subsequent behavioral change with complex messages.

Understanding how self-efficacy shapes the translation of personal norms into environmentally conscious actions offers insights for encouraging sustainable behaviors. Policymakers, media organizations, and non-governmental organizations should consider this influence. The research indicates that a strong belief in one’s ability to take environmentally friendly actions strengthens the relationship with personal norms. Interventions should enhance individuals’ self-efficacy to promote pro-environmental behavior. Tailoring these interventions can increase adherence to personal norms, and communication strategies should highlight personal norms and success, empowering individuals to believe in their capacity to make a positive impact. Educational programs should focus on developing environmental knowledge and skills, boosting self-efficacy and confidence in environmentally friendly actions.

### 6.3 Limitations and future research

This study examined the influence of media and communication factors on PEB through the lens of the norm activation model. By adopting a broad conceptual approach, the study focused on general tendencies toward PEB rather than specific behaviors. While this approach offers a comprehensive view, future research is encouraged to target specific domains of environmentally responsible behavior, such as green purchasing, waste management, or water conservation, for more actionable insights. A notable limitation of this study involves the potential influence of social desirability bias, particularly due to the reliance on self-reported measures to assess personal norms and behavioral intentions. Social desirability bias refers to individuals’ tendency to provide responses that they believe are socially acceptable or favorable, rather than accurate (Paunonen and LeBel, 2012). In this context, participants may have overreported environmentally responsible behaviors or moral commitments to present themselves in a more positive light (Harrison et al., 2023). Such tendencies can inflate estimates of environmental engagement, weaken the validity of findings, and distort the interpretation of relationships among key constructs. This issue may be further amplified in the Taiwanese cultural setting, where environmental responsibility has become a widely emphasized social value. As a result, the pressure to align with perceived social expectations may be even more pronounced, potentially compromising the authenticity of responses. Consequently, the generalizability of the findings to real-world behaviors must be approached with caution. To address this limitation, future research should consider integrating behavioral data or employing indirect measurement techniques, such as experimental designs or anonymous, scenario-based questionnaires, to minimize social desirability effects. Techniques like indirect questioning or the use of behavioral proxies can reduce response bias and enhance the validity and reliability of data collected on sensitive or normative topics.

In terms of cross-cultural generalizability, Taiwan presents a sociocultural context characterized by strong collectivist and Confucian values, which prioritize social harmony, interdependence, and deference to authority. These cultural attributes may amplify the salience of social norms and moral obligations, core components of the NAT, to a greater extent than in more individualistic societies. Additionally, while Taiwan’s media landscape is pluralistic and relatively free, it is also marked by intense competition and pronounced partisanship (Sullivan et al., 2018). This unique media environment may influence the nature and extent of media dependency, a core concept within MSDT, in ways that may not be replicable in countries with state-controlled or less diverse media systems. Furthermore, Taiwan demonstrates relatively robust environmental policies and high levels of civic engagement in environmental issues. Consequently, the generalizability of the findings may be limited in contexts where environmentalism is less institutionalized or is framed through different political lenses. Future research should, therefore, consider cultural norms as a potential moderating variable and seek to replicate the study in more individualistic societies and in countries with weaker environmental policy frameworks.

Future studies should consider longitudinal research to track individuals’ media consumption patterns alongside psychological norm activations, such as awareness of consequences, ascription of responsibility, and personal norms. This would provide insight into how sustained exposure to pro-environmental media messages influences behavioral change over time. Additionally, field experiments that systematically manipulate exposure to media content designed to enhance specific components of the NAT, such as using responsibility framing to strengthen personal norms, could help assess corresponding behavioral outcomes in real-world contexts such as recycling and energy conservation.

## 7 Conclusion

The present study integrates MSDT and NAT theories and F-DEMATEL and SEM approaches to explore the impact of media on environmental communication. Results show that media factors influence awareness of consequences and responsibility ascription. Personal norms mediate the relationship between these factors and PEB. The study also introduces a moderator, self-efficacy, which strengthens the relationship between personal norms and PEB. This research provides insights into how media organizations, policymakers, and non-governmental organizations can enhance pro-environmental actions through various communication strategies.

## Data Availability

The original contributions presented in the study are included in the article/supplementary material, further inquiries can be directed to the corresponding author.
